# Ulcerated Cancer of the Rectum Cured by Extirpation

**Published:** 1829-04-01

**Authors:** 


### HOPITAL DE VERSAILLES.

### ULCERATED "CANCER" OF THE RECTUM CURED BY EXTIRPATION.

Jean Baptiste Legeron, ætatis 30, of bilious temperament and delicate habit, had laboured, during the Spring and Summer of 1828, under obstinate constipation, with a feeling of weight and lancinating pains in the rectum, for which he was forced to employ enemata, and which were thought to depend on a hæmorrhoidal affection. In the month of September, he applied to M. Maurin at the hospital, who found, on introducing the finger into the rectum, an oval, hard, irregular tumour, ulcerated in its centre, and situate on the left side of the gut, about three inches from the anus. A fetid and abundant discharge took place, especially on passing the finger through the sphincter, but the moveable nature of the tumour induced M. Maurin to entertain favourable hopes of its extirpation. The patient was seen on the 17th by M. Dupuytren, who pronounced the tumour carcinomatous, and incurable without an operation, which latter, however, he considered as neither devoid of difficulty or danger. On the 21st, M. Maurin proceeded to operate, confirmed in his opinion by that which had been given by M. Dupuytren.

The patient being placed upon the left side, with the left limb extended and the right flexed, the sphincter was divided by a probe-pointed bistoury, on the finger, to the extent of half an inch, at its left and posterior part. The tumour was then laid hold of, and gradually drawn down through the external opening, when its origin, from the side of the gut, was cautiously cut through with curved scissors. The operation was very painful, and the patient lost a considerable quantity of blood, but the hæmorrhage was stopped by plugging the gut, and did not afterwards return. The tumour, on examination, proved to be oval, a little flattened, ulcerated to the extent of an inch on its surface, two inches long, and compact in texture. It appeared to be developed immediately beneath the mucous membrane of the gut, and the muscular coat had not been wounded in the operation. Some smart febrile symptoms followed the operation, and required one or two abstractions of blood; an abundant suppuration was afterwards established, but gradually decreased in quantity; the pains which had plagued him so much and so long quickly subsided; the wound in the sphincters cicatrized; and, at length, the patient was discharged the hospital completely cured. On examining the rectum, no tumour was found, but only a depression in the part where it had been.—*Journ. Hebdom.*

We really see no reason whatever for concluding that the tumour was of cancerous nature; indeed, it is notorious that the French do not limit the term to the strict acceptation it receives amongst ourselves. It appears to us from the history and general character of the disease, that it might have been originally an internal pile, which ulcerated, as hæmorrhoids often do, and increased to a considerable bulk. We may perhaps be wrong, but under the circumstances detailed we do not think that the tumour was a cancer.

